# Preschoolers Understand the Moral Dimension of Factual Claims

**DOI:** 10.3389/fpsyg.2018.01841

**Published:** 2018-09-28

**Authors:** Emmily Fedra, Marco F. H. Schmidt

**Affiliations:** ^1^International Junior Research Group Developmental Origins of Human Normativity, Department of Psychology, LMU Munich, Munich, Germany; ^2^Department of Developmental and Educational Psychology, University of Bremen, Bremen, Germany

**Keywords:** factual claims, normativity, norm psychology, social-cognitive development, assertive speech acts, moral cognition

## Abstract

Research on children’s developing moral cognition has mostly focused on their evaluation of, and reasoning about, others’ intrinsically harmful (non-)verbal actions (e.g., hitting, lying). But assertions may have morally relevant (intended or unintended) consequences, too. For instance, if someone wrongly claims that “This water is clean!,” such an incorrect representation of reality may have harmful consequences to others. In two experiments, we investigated preschoolers’ evaluation of others’ morally relevant factual claims. In Experiment 1, children witnessed a puppet making incorrect assertions that would lead to harm or to no harm. In Experiment 2, incorrect assertions would always lead to harm, but the puppet either intended the harm to occur or not. Children evaluated the puppet’s factual claim more negatively when they anticipated harmful versus harmless consequences (Experiment 1) and when the puppet’s intention was bad versus good over and above harmful consequences (Experiment 2). These findings suggest that preschoolers’ normative understanding is not limited to evaluating others’ intrinsically harmful transgressions but also entails an appreciation of the morally relevant consequences of, and intentions underlying, others’ factual claims.

People make assertions about the world every day. Many of these (e.g., “The sun is smaller than the earth.") are typically orthogonal to moral issues and can simply be accepted or rejected given observable reality or some piece of evidence. Others may be morally relevant for a speaker intends to (interpersonally) deceive an addressee (e.g., lying). But sometimes, even simple factual claims – which we keep distinct from the term “lying" – (e.g., “This water is clean!") may become morally relevant, in that they may have harmful consequences (e.g., influence others to act in harmful ways). What is more, speakers may even use factual claims that are easily refutable (e.g., simple generalizations, or claims like “This project was not a success!," “The Earth is flat!") not so much to deceive others, but rather as a means to bring about certain (harmful) consequences (e.g., instill conflict, uncertainty). That is, factual claims may have a moral dimension over and above questions of deceptive intent, truthfulness (i.e., whether the speaker believes the claim or not), or intrinsic harmfulness (e.g., insults). Perhaps especially in the digital age of today in which we face all kinds of assertions that may be associated with certain (intended or unintended) consequences, it seems vital to assess children’s understanding of the moral relevance of simple factual claims. In the present study, we investigate preschoolers’ understanding of the moral dimension of others’ factual claims with a focus on harmful consequences on the one hand, and harmful intentions (regarding harmful consequences) on the other.

## Children’s Evaluation of Others’ Moral Transgressions

Developmental research over the past couple of decades has accumulated much evidence that preschoolers and, to some extent, even very young children understand much about the moral dimension of others’ actions (Turiel, 2006; Schmidt and Tomasello, 2012; Hamlin, 2013; Killen and Smetana, 2015; Rottman and Young, 2015; Sommerville and Enright, 2018). Most prominently, a bulk of interview studies based on social domain theory suggests that preschoolers reliably differentiate between moral norms (e.g., norms forbidding violent behavior, such as hitting) and conventional norms (e.g., norms prescribing appropriate clothing, such as not wearing pajamas to school), judging that – compared with conventional violations – moral transgressions are more severe, more deserving of punishment, more widely applicable and independent of authority demands (Turiel, 2006; Smetana et al., 2014; Killen and Smetana, 2015). Another line of research focused on children’s disinterested enforcement of norms in social interactions and found that from around 3 years of age, children spontaneously protest and criticize agents who violate conventional norms, such as (agreed-upon) simple game rules (Rakoczy, 2008; Rakoczy et al., 2008; Schmidt et al., 2016), and agents who commit moral transgressions, such as violating others’ rights or harming others (Rossano et al., 2011; Vaish et al., 2011; Schmidt et al., 2012, 2013).

And from around 3 to 5 years of age, children do not just reject and negatively evaluate harmful physical actions but also show some understanding of intrinsically harmful verbal actions that produce psychological harm (typically given the content of the speech act), such as name-calling or teasing (Helwig et al., 2001; Smetana et al., 2012; Ball et al., 2017), or “epistemic harm” (given the speaker’s deceptive intent to instill a false belief in the listener), such as lying and deceiving (Peterson et al., 1983; Bussey, 1999; Lyon et al., 2013). Together, these studies using different methodologies equally suggest that at preschool age, children understand much about the moral dimension of intrinsically harmful non-verbal and verbal actions.

## Children’s Evaluation of Others’ Assertions

While there is much evidence that preschoolers understand the moral dimension of others’ intrinsically harmful (non-)verbal actions (e.g., hitting, stealing, lying), there is, to our knowledge, no research on their understanding (in terms of normative evaluation) of the moral dimension of others’ factual claims that become morally relevant not because of their deceptive motivation, but because of the harmful consequences – intended or not – they may entail. Past work has focused on whether children, or even infants, categorize others’ speech acts as correct or incorrect or, at minimum, as statistically expectable or not. For instance, research has shown that even infants are sensitive to whether a speaker labels an object correctly (Koenig and Echols, 2003). And 2-year-olds spontaneously reject assertions that do not match reality (e.g., “Peter is eating the cake” when Peter instead is eating a carrot; Pea, 1982). Moreover, 3-year-olds understand that imperative speech acts should lead to a change of reality (e.g., a person should follow an imperative), whereas assertive speech acts should describe the present reality correctly (Rakoczy and Tomasello, 2009). At the same age, children can identify persons that say something correct or say something wrong and distinguish correct from incorrect statements (Koenig et al., 2004; Lyon et al., 2013).

## Investigating Children’s Understanding of the Moral Dimension of Factual Claims

Some assertions, such as (malicious) lies, may be considered intrinsically harmful as they are morally relevant regardless of their consequences (Turiel, 1983; Lee, 2013). That is, even if a lie is not effective or does not produce major harm, we may find the mere act of lying, the deceptive intent, blameworthy. However, there is also a more extrinsic component of moral relevance to assertions, namely, the potentially harmful consequences they may entail. For instance, factual claims, such as “This water is clean!,” may simply be false given observable reality. Thus, one may easily refute them. However, they may also bring about harmful consequences beyond questions of truthfulness or deception (e.g., someone might get sick by drinking dirty water). Thus, we can morally evaluate assertions for their consequences just like physical actions (Cushman, 2008). Moreover, we may have information about whether the speaker intends harmful consequences to occur or not. Importantly, the speaker may not even have deceptive intent or believe the claim to be false, but rather use the speech act to bring about harmful consequences. Thus, we may also morally evaluate assertions for the intentionality of their consequences.

Hence, here we are interested in two major questions concerning children’s understanding of the moral dimension of factual claims: (i) how do children evaluate assertions that lead to harmful consequences? And (ii) does it matter for children’s moral evaluation whether the harmful consequences were intended by the speaker or not? Evaluating morally relevant assertions is more complex than evaluating morally relevant actions. Regarding the former, the child can directly assess someone’s action considering moral norms or principles (e.g., “Hitting is wrong!”). Regarding the latter, however, the child needs to infer that a factual claim (e.g., “This is an X!”) – which, *per se*, could be considered amoral in that it merely corresponds to reality or not (Turri, 2017) – may lead to harmful consequences and that those consequences may be intended or not. Hence, the crux is to evaluate the assertion as good or bad not in light of its correspondence to reality, but regarding the *moral relevance* of its *consequences* and the *intentionality* of those consequences.

Ever since Piaget (1932) seminal work, researchers were interested in whether children put more weight on the consequences of an agent’s morally relevant action or on the agent’s mental states, such as intention, when evaluating the moral valence of an act. While Piaget was clear that children begin with outcome-based evaluations and only later consider others’ intentions in their moral evaluation, more recent research produced heterogeneous results. Whereas some researchers suggest that even school-aged children tend to give more weight to outcomes than to intentions (Costanzo et al., 1973; Yuill, 1984; Zelazo et al., 1996; Helwig et al., 2001; Cushman et al., 2013; Gummerum and Chu, 2014), others found that when using simplified procedures (e.g., simpler vignettes) or controlling for confounding factors (e.g., the action of the well-intended and the ill-intended actors led to the same outcome), even 4- to 5-year-old (and in some work, even 3-year-old) children consider an agent’s intention (Chandler et al., 1973; Nelson, 1980; Baird and Astington, 2004; Nobes et al., 2009, 2016; Vaish et al., 2010; Killen et al., 2011; Gvozdic et al., 2016). A recent study (Josephs et al., 2016) demonstrated that 4-year-old (and to some extent even 3-year-old) children take into account an agent’s intentionality (freedom of choice) and protested more when a moral transgression occurred under free conditions than if it occurred under constrained ones. For conventional violations, however, children tended to put more weight on outcomes.

When evaluating others’ morally relevant factual claims, children thus need to coordinate both consequences (e.g., harmful vs. harmless) and intentions (e.g., good vs. bad) regarding consequences. For intentions, in particular, children are required to use both their normativity and theory of mind skills (Perner et al., 1989; Killen and Smetana, 2008; Killen and Rizzo, 2014; Schmidt and Rakoczy, 2018).When it comes to explicitly evaluating others’ morally relevant actions, children begin to consider the importance of intentions by around 4 to 5 years of age (Nelson, 1980; Nobes et al., 2016), which coincides with children’s becoming competent at false belief tasks (Perner and Roessler, 2012). Recently, Killen and colleagues (2011) investigated 3.5- to 7.5-year-old children’s understanding of intentions in a morally relevant context – morally relevant theory of mind (MoToM). In MoTom tasks, children receive vignettes in which a “transgressor” accidentally causes harm to another person (e.g., accidentally throws a bag with another person’s cupcake away). Children who failed classical false belief tasks were more likely to attribute bad intentions to an accidental transgressor and to accept punishment of the accidental transgressor than children who passed the false belief task. Overall, children began to take into account the transgressor’s intention between 3.5 and 5.5 years of age.

## The Present Study

In the current study, therefore, we are interested in speech acts that are in and of themselves amoral (i.e., they are simply correct or incorrect and not deceptive), but come with moral relevance, either in terms of anticipated consequences or in terms of the intentionality of those consequences. We sought to investigate in two experiments whether 4- to 5-year-old children understand the moral dimension of factual claims and evaluate and reason about such claims in terms of morally relevant consequences (Experiment 1) or the intentionality of morally relevant consequences (Experiment 2). Importantly, to investigate children’s evaluation of assertions, and not of (non-verbal) actions, one needs to make sure that children only witness a speaker making an assertion, but not performing an action (which could be directly assessed without referring to the speaker’s assertion). Moreover, to exclude the moral evaluation of epistemic harm (e.g., deceptive intent) and psychological harm (e.g., teasing), it is crucial to use assertions that can easily be rejected given observable reality, and that do not have a specific addressee (that might be deceived or insulted). In Experiment 1, therefore, a puppet made simple incorrect factual claims (e.g., “This is an X!,” although it was a Y) and children were told that this incorrect claim would either lead to harm (another puppet would lose her property) or to no harm (a paper ball would be thrown away). In Experiment 2, incorrect claims would always lead to harm, but the puppet either intended the harmful consequences (bad intention) or not (good intention). We predicted that preschoolers would evaluate the incorrect factual claim more negatively (i) when it would lead to harm than when it would not cause any harm (Experiment 1), and (ii) when it was based on a bad intention than when its underlying intention was good (Experiment 2). Moreover, we predicted that children who differentiate correctly between the two types of incorrect factual claims in both experiments would be more likely to provide adequate justifications (referring to consequences in Experiment 1, and to intentions in Experiment 2) for their differential evaluation than children who did not differentiate between the two types of incorrect factual claims.

## Experiment 1

In Experiment 1, we sought to investigate how children evaluate and justify their evaluation about others’ morally relevant factual claims. We manipulated the consequences of the incorrect claim: it would either lead to harm or to no harm.

### Methods

#### Participants

Twenty-four (51–69 months; *M* = 5 years, 0 months; 12 girls) preschoolers participated in the study. Children came from mixed socio-economic backgrounds from a large German city and were recruited *via* urban daycare centers (in which testing took place). Parents provided written informed consent. One additional child was tested but excluded due to uncooperativeness.

#### Design

In a within-participants design, all children received a factual claim task with two conditions: a puppet made an incorrect claim that would either lead to harm (harm condition) and to no harm (no harm condition). The factual claim task was preceded by a warm-up session (playing with a ball) and a training phase which consisted of two instrumental warm-up tasks (one harm, one no harm condition). The order of condition was counterbalanced between children.

#### Procedure

Two experimenters conducted the study, which lasted roughly 10 minutes: E1, the coordinator, and E2, who operated two puppets (an elephant named “Susi” and an owl named “Lore”). The child, E1, and E2 sat at a table. E1 sat to the child’s left, and E2 on the child’s right. The factual claim task was preceded by a training phase with two warm-up tasks to make sure children understood the consequences of an incorrect behavior that led either to harm or to no harm.

##### Training phase

In the harm condition, E1 first showed the child and the puppets five stickers and put them in front of the owl (“Look Lore, these are your stickers. These are yours. Look [referring to the child] these are Lore’s stickers and Lore really likes these stickers.”). The owl confirmed this by saying, “Yes, I really like these stickers! And if my stickers are gone, I will be very sad!” and subsequently said goodbye and went to sleep. First, the experimenter performed an instrumental action that the child could reproduce (e.g., using a hammer to hit on wooden balls to send them through holes of a cuboid). After that she put a box on the table asking the child to pay attention (“And now pay attention to what Susi will do! But Susi must not do anything wrong! If Susi does something wrong, I will take away all of Lore’s stickers and put them in this box and then Lore is very sad!”). In the no harm condition, there was only the elephant present and instead of stickers, a paper ball was the object of interest. The experimenter showed the child another instrumental action that the child could reproduce (e.g., putting a disc on a peg). Thereafter, the experimenter put a box on the table asking the child to pay attention (“And now pay attention to what Susi will do! But Susi must not do anything wrong! If Susi does something wrong, I will take this paper and put it in this box and then no one is sad!”). In the test phase of both the harm and the no harm conditions, the elephant made an instrumental mistake by failing to use a conventional means necessary to achieve an aim (e.g., failing to use the hammer). When the experimenter turned back she asked the child two control questions, “Did Susi do it right or wrong?” and “What will I do with these stickers/the paper?” Depending on the child’s answer, the experimenter either confirmed the child’s answer or she corrected him/her, and as announced, the experimenter put the stickers/paper in the box on the table. After answering the control questions, the child was asked to evaluate the elephant’s action for its moral valence on a four-point Likert scale with smiley faces as anchor (“Susi did it wrong. Is this very bad [German: “schlecht”], a little bad, good or very good.”) and was asked to justify his/her evaluation.

##### Factual claim task

The important difference between the factual claim task and the warm-up tasks in the training phase was that instead of evaluating instrumental actions the child was asked to evaluate factual claims for their moral valence and the child did not see the announced consequences, but had to anticipate them. The setup was similar to the one in the training phase but differed in two ways: in the harm condition, the stickers were replaced by gems and in both conditions, objects were used instead of toys. In the introduction phase, the owl again declared that she likes her gems very much and would be very sad if her gems would be gone and subsequently went to sleep. Then, the experimenter put an object (e.g., a spoon) and a box on the table and asked the child to pay attention to what the elephant was going to say “And now pay attention to what Susi will say. But Susi must not say anything wrong! If Susi says something wrong, I will take away all of Lore’s gems and put them in this box and then Lore is very sad.” (*harm condition*), or “If Susi is saying something wrong, I will take this paper and put it in this box and then no one is sad!” (*no harm condition*). When the experimenter had turned around, the elephant thought aloud: “Well, when I am saying something wrong, [experimenter’s name] will take away all of Lore’s gems and put them in this box and then Lore is very sad.” (harm condition), or “Well, when I am saying something wrong, [experimenter’s name] will take this paper and put it in this box and then no one is sad!” (no harm condition).

In the test phase of both conditions, the elephant pointed to the object (e.g., spoon) and made an incorrect claim: “I say this is an X (e.g., cat).” The experimenter then turned back and corrected the elephant saying, “This is an Y, not an X!” The child was then asked to evaluate the elephant’s speech act for its moral valence on a four-point Likert scale with smiley faces as anchor (“Susi said it wrong. Is this very bad, a little bad, good or very good?”) and to justify his/her evaluation.

#### Coding and Reliability

All sessions were transcribed and coded from videotape by a single observer. A second independent observer, blind to the hypotheses and conditions of the study, transcribed and coded a random sample of 25% of all sessions for reliability.

Children’s answers to the control questions (dichotomous variable: correct or incorrect response to E1’s questions), their evaluation on the Likert scale – from 1 (very good) to 4 (very bad) – and the justification of their evaluation were coded. Children’s verbal responses were assigned to the following categories (the first and third categories were determined a priori; see also Nobes et al., 2009): (a) references to consequences (e.g., “Because now all gems are gone.”; “Because now no one is sad.”); (b) references to the elephant’s actions and speech acts (e.g., “Because she did it wrong.,” “Because it is not a cat.”); (c) references to the elephant’s intentions (e.g., “Because she [the elephant] wants to have the stickers.”); (d) irrelevant justifications (e.g., “Because the gems are so beautiful.”); or (e) no justifications (including “Don’t know”).

Interrater reliability was very good, Cohen’s κ = 1 (both answers to the control question 1 and 2), κ = 1 (warm-up task evaluation), κ = 1 (warm-up task justification), κ = 1 (factual claim task evaluation), and κ = 1 (factual claim task justification).

#### Statistical Analysis

Statistical Analysis were run in R, version 3.4.2 (R Core Team, 2016). For the measure evaluation of the action in the warm-up and the speech act in the factual claim task, we used non-parametric statistics (Wilcoxon *Z*-tests) instead of paired sample *t*-tests, because errors were not normally distributed. For non-parametric tests, we computed the generic effect size *r*.

### Results

#### Factual Claim Task

##### Evaluation

**FIGURE 1:**
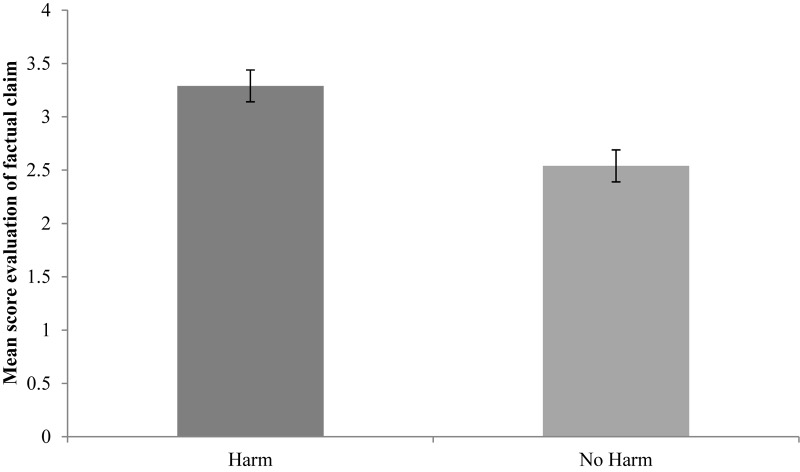
Mean score of children’s evaluation (from 0 = very good to 4 = very bad). Error bars depict standard error of the mean.

In the factual claim task, children evaluated the puppet’s speech act significantly more negative when the speech act would lead to harm (*M* = 3.29, *SD* = 0.75) than when it would lead to no harm (*M* = 2.54, *SD* = 1.06; *Z* = −2.360, *p* = 0.018, *r* = 0.481). **Figure 1** shows the mean score of children’s evaluation of the puppet’s speech act.

##### Justifications

Children also had the opportunity to justify their evaluation. **Table 1** shows the frequencies of children’s justifications.

**Table 1:** Frequencies (percentage) of justifications.

	Task			
Category	Warm-up		Factual claim	
	Harm	No harm	Harm	No harm
Consequences	6 (25%)	3 (12.5%)	7 (29%)	6 (25%)
Action/speech act	10 (42%)	12 (50%)	11 (46%)	10 (42%)
Intentions	1 (4%)	0	0	0
Others	2 (8%)	3 (12.5%)	2 (8%)	2 (8%)
No answer	5 (21%)	6 (25%)	4 (17%)	6 (25%)

##### Relation between evaluation and justifications

For the purposes of analyses, children were categorized as “*competent*” (i.e., children who evaluated the puppet’s speech act that would lead to harm more negatively than the speech act that would lead to no harm) and “*other”* (i.e., the rest of the sample). There were significant associations between children’s justifications and their competence in evaluating the moral valence of the puppet’s speech act both when it would lead to harm, χ^2^ (2, *N* = 24) = 6.45, *p* = 0.011, *V* = 0.42 and to no harm, χ^2^ (2, *N* = 24) = 4, *p* = 0.045, *V* = 0.31 (see **Table 2**), such that competent children were more likely to justify their evaluation referring to the consequences of the speech act (rather than using other types of justification) than other children.

**Table 2:** Association between evaluation and justification.

				Justification category		
Task	Condition			Intentions	Consequences	Others
Factual claim	Harm	Evaluation	Others	0	2	14
			Competent	0	5	3
	No harm	Evaluation	Others	0	2	14
			Competent	0	4	4
Warm-up	Harm	Evaluation	Others	0	4	11
			Competent	1	2	6
	No harm	Evaluation	Others	0	0	15
			Competent	0	3	6

#### Warm-Up Task

Children answered two control questions in the warm-up tasks to make sure they understood the consequences of a wrong action. In the harm condition, one child (4%), and in the no harm condition, two children (8%) gave incorrect answers to the first control question (“Did Lore do it right or wrong?,” correct answer was “wrong”). In the harm condition, no child, and in the no harm condition, two children (8%) gave an incorrect answer to the second control question (“And what will I do with the stickers/paper?,” correct answer was “You put them/it in the box.”).

##### Evaluation

In the warm-up tasks, children evaluated the wrong behavior significantly more negative when the action led to harm (*M* = 3.38, *SD* = 0.65) than when it led to no harm (*M* = 2.71, *SD* = 1; *Z* = −2.495, *p* = 0.011, *r* = 0.509).

##### Justifications

See **Table 1** for the frequencies of children’s justifications.

##### Relation between evaluation and justifications

There was no significant association between children’s justifications and their competence in evaluating the moral valence of the puppet’s action that led to harm, χ^2^ (2, *N* = 24) = 1.74, *p* = 0.587, *V* = 0.27 (see **Table 2**). However, there was a significant association between children’s justifications and their competence in evaluating the moral valence of the puppet’s action that led to no harm, χ^2^ (2, *N* = 24) = 5.71, *p* = 0.016, *V* = 0.36, such that competent children were more likely to justify their evaluation referring to the consequences of the action (rather than using other types of justification) than the other children.

### Discussion

Children in this experiment evaluated the puppet’s factual claim act more negatively when it would lead to harmful consequences than when it would lead to no harm. Moreover, those children who evaluated the puppet’s assertions competently (i.e., evaluating the harm-related assertion as worse than the no harm-related assertion) were more likely to justify their evaluation referring to the consequences of the factual claim than to give irrelevant or no justification, whereas the other children (i.e., those who did not differentiate between the two types of factual claims or gave a more negative evaluation of the no harm-related assertion) were more likely to refer to the incorrect factual claim itself, to give an irrelevant answer or no justification. This suggests that preschoolers’ normative understanding goes beyond evaluating others’ intrinsically harmful (non-)verbal actions (e.g., hitting, lying), and also entails an appreciation of the moral consequences of others’ assertive speech acts. However, this experiment leaves open the question of whether children appreciate morally relevant intentions underlying others’ assertive speech acts when controlling for outcome. Thus, to assess this question, we conducted a second experiment in which consequences would always be harmful and either intended by a puppet (bad intention) or not (good intention).

## Experiment 2

In Experiment 2, in contrast to Experiment 1, incorrect factual claims always would lead to harm. However, the puppet either intended those harmful consequences or not. Findings from different studies suggest that when confronted with vignettes about different types of transgressions, children can differentiate between acts based on good and acts based on bad intentions from around 4 to 5 years of age (Núñez and Harris, 1998; Nobes et al., 2016, 2009). Furthermore, Killen et al. (2011) found that children began to take into account a transgressor’s intention between 3.5 and 5.5 years, such that children who passed classical false belief tasks were more likely to attribute good intentions to an accidental transgressor and to decline punishment of the accidental transgressor than children who failed the false belief task. Importantly, we went beyond prior work and did not investigate whether children consider intentions when evaluating intrinsically harmful *non-verbal actions* (e.g., physical harm, such as breaking cups or hurting another person accidentally or intentionally) or verbal actions (e.g., lying), but rather whether children consider whether a puppet intends harm to occur when evaluating her speech act. If they do, children should evaluate the well-intended puppet’s incorrect factual claim more positively than the ill-intended puppet’s incorrect factual claim.

### Methods

#### Participants

Twenty-four (48–71 months; *M* = 5 years, 0 months; 12 girls) preschoolers participated in the study. Children came from mixed socio-economic backgrounds from a large German city and were recruited *via* urban daycare centers and a museum (in which testing took place). Parents provided written informed consent. One additional child was tested but excluded due to language difficulties.

#### Design

In a within-participants design, all children received a factual claim task in which a puppet made an incorrect assertion that would always lead to harm. The task had two conditions which differed in that the puppet’s intention was either good or bad (*good-intention condition* and *bad-intention condition*). The factual claim task was preceded by a warm-up session (playing with a ball) and warm-up tasks which consisted of two instrumental tasks. A forced choice task always came last. The order of condition was counterbalanced between children. The order of the puppets’ appearance remained the same (elephant, dog, lion, and seal).

#### Procedure

Two experimenters conducted the study, which lasted roughly 15 minutes: E1, the coordinator, and E2, who operated the victim (an owl puppet), the two actor puppets (an elephant and a dog) and the two speaker puppets (a lion and a seal). Each puppet was used in one trial only. The child, E1, and E2 sat at a table. E1 sat to the child’s left, and E2 sat vis-à-vis to the child (thus the child faced the puppets).

The factual claim task was preceded by a training phase, consisting of two warm-up tasks to make sure children understood the consequences of an incorrect behavior that was based on good or bad intentions.

##### Training phase

E1 first showed the child and the two puppets (e.g., owl and elephant) five stickers and put them in front of the owl (“Look owl, these are your stickers. These are yours. Look [referring to the child] these are the owl’s stickers and she really likes these stickers.”). The owl confirmed this by saying “Yes, I really like these stickers! And if my stickers are gone, I am very sad!” and subsequently said goodbye and went to sleep. First, the experimenter performed an instrumental action that the child could reproduce (e.g., using a hammer to hit on wooden balls to send them through holes of a cuboid). After that she put a box in front of the elephant, and asked the child to pay attention (“And now pay attention to what the elephant will do! But he must not do anything wrong! If he does something wrong, I will take away all of the owl’s stickers and put them in the elephant’s box and then the owl is very sad!”). When the experimenter had turned around, the elephant repeated: “Well, if I do something wrong, [experimenter’s name] will take away all of the owl’s stickers and put them in my box, and then the owl is very sad.” In the bad intention condition, he announced: “The owl should not keep the stickers. I want those stickers. That’s why I want to do something wrong.,” while announcing in the good intention condition: “The owl should keep the stickers. I do not want those stickers. That’s why I want to do something right.”

In the test phase, in both the good and the bad intention conditions, the elephant made an instrumental mistake, by failing to use a conventional means necessary to achieve an aim (e.g., failing to use the hammer). When the experimenter turned back, she asked the child “Did he do it right or wrong?” and “What will I do with these stickers?” Depending on the child’s answer, the experimenter either confirmed the child’s answer or she corrected him/her, and as announced, the experimenter put the stickers in the other puppet’s box. After answering the control questions, the child had to evaluate the elephant’s action for its moral valence on a Likert scale (“The elephant did it wrong. Is this mean [German “böse”], a little mean, good or very good of him?”) and was asked to justify his/her evaluation. Note that we used the German word “böse” to allow children to focus on intentions and not only on the fact that harm occurred or even that the speech act was incorrect.

##### Factual claim task

The important difference between the warm-up task and the factual claim task was that instead of evaluating an instrumental action the child had to evaluate factual claims for their moral valence, and the child did not see the announced consequences, but had to anticipate them. The setup was similar to the one in the warm-up task and differed only in two ways: the stickers were replaced by gems and in both conditions, objects were used instead of toys. In the introduction phase, the owl again declared that she likes her gems very much and would be very sad if her gems would be gone and subsequently went to sleep. Then, the experimenter put a box in front of the speaker puppet (e.g., the lion) and an object (e.g., a spoon) on the table, and asked the child to pay attention to what the speaker puppet was going to say (“And now pay attention to what the lion will say. But he must not say anything wrong! If he says something wrong, I will take away all of the owl’s gems and put them in the lion’s box and then the owl is very sad.”). When the experimenter had turned around, the speaker puppet repeated: “Well, when I am saying something wrong, [experimenter’s name] will take away all of the owl’s gems and put them in my box and then the owl is very sad.” In the bad intention condition, the puppet announced: “The owl should not keep the gems. I want those gems. That’s why I want to say something wrong.,” while announcing in the good-intention condition: “The owl should keep the gems. I do not want those gems. That’s why I want to say something right.”

In the test phase of both conditions, the speaker puppet pointed on the object (e.g., spoon) and made an incorrect claim: “I say this is an X (e.g., cat).” The experimenter then turned back and corrected the lion (“This is an Y, not an X!”). The child was asked to evaluate the lion’s claim for its moral valence on a Likert scale (“The elephant said it wrong. Is this mean, a little mean, good or very good of him?”) and to justify his/her evaluation.

After the evaluation trials, both speaker puppets (lion and seal) who took part in the factual claim task came back. The experimenter repeated the puppets’ intentions: “The lion wanted to have the owl’s gems and therefore wanted to say something wrong. And the seal did not want to have the owl’s gems and therefore wanted to say something right. And then both said something wrong. But who of the two is mean?” The child had to choose one puppet and was asked to justify his/her choice.

#### Coding and Reliability

All sessions were transcribed and coded from videotape by a single observer. A second independent observer, blind to the hypotheses and conditions of the study, transcribed and coded a random sample of 25% of all sessions for reliability.

Children’s answers to the control questions (dichotomous variable: correct or incorrect response to E1’s questions), their rating on the Likert scale – from 1 (very good) to 4 (mean) –and the justification of their rating were coded. Children’s verbal responses were assigned to categories: (a) references to the puppet’s intention (e.g., “Because he did it on purpose.,” “Because he said he wants to say it right); (b) references to the consequences (e.g., “Because now all gems are gone.,” “Because then she [the owl] is sad.”); (c) references to the puppet’s action or claim (e.g., “Because he did it wrong.,” “Because they are actually scissors.”); (d) references to the ownership (e.g., “Because these are the owl’s gems.”); (e) irrelevant justifications (e.g., “Because he has sharp teeth.”); or (f) no justifications (including “Don’t know”).

Interrater reliability was very good, Cohen’s κ = 1 (both answers to the control question 1 and 2), Cohen’s κ = 1 (warm-up task evaluation), Cohen’s κ = 1 (warm-up task justification), Cohen’s κ = 1 (factual claim task evaluation), Cohen’s κ = 1 (factual claim task justification), Cohen’s κ = 1 (forced-choice task : “Who of the two is mean?”), Cohen’s κ = 1 (forced-choice task justification).

#### Statistical Analysis

Statistical Analysis were run in R, version 3.4.2 (R Core Team, 2016). Analyses were carried out as in Experiment 1.

### Results

#### Factual Claim Task

##### Evaluation

**FIGURE 2:**
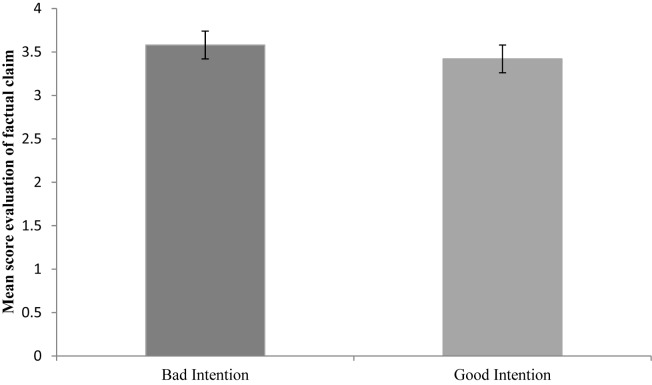
Mean score of children’s evaluation (from 0 = very good to 4 = mean). Error bars depict standard error of the mean.

In the factual claim task, children evaluated the puppet’s speech act significantly more negatively when the puppet’s intention was bad (*M* = 3.58, *SD* = 0.78) than when it was good (*M* = 3.42, *SD* = 0.78; *Z* = −2.00, *p* = 0.046, *r* = −0.408). **Figure 2** shows the mean score of children’s evaluation of the puppet’s speech act.

##### Justifications

Children also had the opportunity to justify their evaluation. **Table 3** shows the frequencies of children’s justifications.

**Table 3:** Frequencies (percentage) of justifications.

	Task			
Categories	Warm-up		Factual claim	
	Bad intention	Good intention	Bad intention	Good intention
Bad intention	5 (21%)	0	6 (25%)	1 (4%)
Good intention	0	4 (17%)	0	3 (12.5%)
Consequences	5 (21%)	4 (17%)	5 (21%)	5 (21%)
Action/speech act	10 (42%)	11 (46%)	10 (42%)	10 (42%)
Ownership	0	1 (4%)	1 (4%)	1 (4%)
Others	1 (4%)	1 (4%)	1 (4%)	1 (4%)
No answer	3 (12%)	3 (12%)	1 (4%)	3 (12.5%)

##### Relation between evaluation and justification

For the purposes of analyses, children were categorized as “*competent*” (i.e., children who evaluated the puppet’s speech act that was based on bad intentions more negatively than when it was based on good intention) and “*other*” (i.e., did not differentiate between the two conditions). As predicted, there were significant associations between children’s justifications and their competence in evaluating the moral valence of the puppet’s speech act (see **Table 4**): bad intentions, χ^2^(2, *N* = 24) = 14.40, *p* = 0.001, *V* = 0.775; good intentions, χ^2^(2, *N* = 24) = 11.88, *p* = 0.002, *V* = 0.703, such that children who evaluated the puppet’s speech act competently were more likely to give justifications that referred to the puppet’s intentions (rather than using other justification categories) than children who did not differentiate between the two puppets. These children were more likely to give justifications that referred to the consequences of the speech act, irrelevant justifications or no justifications.

**Table 4:** Association evaluation and justification.

				Justification category		
Task	Intention			Intentions	Consequences	Others
Factual claim	Bad	Evaluation	Others	2	5	13
			Competent	4	0	0
	Good	Evaluation	Others	1	5	14
			Competent	3	0	1
Warm-up	Bad	Evaluation	Others	1	4	14
			Competent	4	1	0
	Good	Evaluation	Others	1	4	14
			Competent	3	0	2

#### Forced-Choice Task

After the evaluation phase, children were asked to identify the “mean” puppet. To test whether the proportion of children choosing correctly the puppet with bad intentions was significantly different from chance (0.50), we conducted a planned exact binomial test (two-tailed). Children reliably chose the puppet with bad intentions (88% of children, *p* < 0.001). Furthermore, children were asked to justify their choice. Of the children who correctly identified the ill-intended puppet as the “mean” (German: “böse”) puppet, nine children (43%) referred to the puppet’s bad intentions, three children (14%) to the wrong speech act, two children (10%) to the consequences in their justification, three children (14%) gave an irrelevant, and four children (19%) gave no justification. Of the children who incorrectly identified the well-intended puppet as the “mean” puppet, one child referred to the puppet’s bad intentions (33%), one child (33%) to the puppet’s good intentions in their justification, and one child (33%) gave an irrelevant justification.

#### Warm-up Task

In the warm-up task, children answered two control questions to make sure they understood the consequences of a wrong action based on good or bad intentions. Only when the puppet had good intentions, eight children gave an incorrect answer to the first control question (“Did she do it right or wrong?,” correct answer was “wrong”). When the puppet had bad intentions, one child gave an incorrect answer to the second control question (“And what will I do with the stickers?,” correct answer was “You put them in the puppet’s box.”).

##### Evaluation

In the training phase, children evaluated the puppet’s action marginally more negative when the puppet had bad intentions (*M* = 3.62, *SD* = 0.71) than when she had good ones (*M* = 3.42, *SD* = 0.78; *Z* = −1.67, *p* = 0.096., *r* = −0.340).

##### Justifications

See **Table 3** for the frequencies of children’s justifications.

##### Relation between evaluation and justification

As predicted, there were significant associations between children’s justifications and their competence in evaluating the moral valence of the puppet’s action (see **Table 4**): bad intentions, χ^2^(2, *N* = 24) = 14.29, *p* = 0.001, *V* = 0.772; good intentions, χ^2^(2, *N* = 24) = 8.84, *p* = 0.012, *V* = 0.607, such that children who evaluated the puppet’s action competently were more likely to give justifications that referred to the puppet’s intentions (rather than using other justification categories) than children who did not differentiate between the two puppets or wrongly evaluated the puppet’s action more negatively when it was based on good than on bad intentions. These children were more likely to give justifications that referred to the consequences of the action, irrelevant justifications or no justifications.

### Discussion

Children in this experiment evaluated the puppet’s factual claim – which was always incorrect and would always lead to harm – more negatively when the puppet intended the harmful outcome (bad intention) than when the puppet did not intend the harmful outcome (good intention). Moreover, competent children (who evaluated the ill-intended speech act more negatively than the well-intended one) were more likely to give justifications that referred to the puppet’s intentions than to the consequences of the assertive speech act, whereas the other children (i.e., who did not distinguish between the two speech acts) were more likely to give a justification that referred to the consequences of the speech act, or, for instance, to the wrong speech act itself than to the puppet’s intention. Furthermore, children reliably chose the ill-intentioned puppet as being the “mean” puppet. These findings suggest that preschoolers’ normative understanding of morally relevant assertions also entails an appreciation of the intentions underlying those speech acts.

## General Discussion

Much developmental research on children’s understanding of normativity and morality focused on their evaluation of others’ intrinsically harmful (non-)verbal actions, such as hitting, stealing, lying, or teasing. Verbal actions (e.g., assertions), however, may have a moral dimension beyond epistemic harm (e.g., lying) or psychological harm (e.g., teasing). For instance, if someone makes an incorrect factual claim (e.g., “This water is clean!” or “The Earth is flat!”), this may lead to harmful consequences to others. And the speaker may even want those harmful consequences to occur and therefore misuse the factual claim to reach an ill-intended goal. We investigated children’s understanding of the moral dimension of factual claims. In two experiments, children witnessed a speaker making an incorrect assertion (“This is an X!”). In Experiment 1, we varied the speech act’s consequences: it would either lead to harm (another puppet would lose her property) or to no harm (a paper ball would be thrown away). Children evaluated the incorrect factual claim that would lead to harm more negatively than the incorrect factual claim that would not lead to any harm. In Experiment 2, the incorrect assertion would always lead to harm (a puppet would lose her property). However, we varied whether the puppet’s intention was good (harmful consequences were not intended) or bad (harmful consequences were intended). When the speaker was ill-intended, children evaluated her claim more negatively than when she was well-intentioned, although both claims would lead to harmful consequences. Importantly, in neither experiment did children witness morally relevant (non-verbal) actions in the factual claim task, such as throwing away someone’s property. Rather, they witnessed and evaluated morally relevant factual claims that were related to upcoming consequences or prior intentions.

These findings go beyond previous work on children’s evaluation of, and reasoning about, others’ morally relevant (non-)verbal actions (e.g., hitting, stealing, lying, and teasing) in interview studies (Peterson et al., 1983; Turiel, 1983; Tisak and Turiel, 1988; Bussey, 1992; Smetana, 2006; Smetana et al., 2012) and children’s spontaneous protest responses to norm transgressions in social interactions (Schmidt and Tomasello, 2012). In our study, children did not witness concrete harming non-verbal actions, psychological harm or epistemic harm, but rather factual claims (which, *per se*, need not be considered moral, but rather correct or incorrect given observable reality; Turri, 2017) with moral relevance. Our findings also go beyond prior work on preschoolers’ evaluation of speech acts which did not involve a moral dimension, such as harm. For instance, 3-year-olds were found to criticize speakers who make incorrect factual claims (Rakoczy and Tomasello, 2009). In our experiments, however, claims were always incorrect, and children had to reason about the additional moral layer (consequences or intentionality of consequences) when evaluating the factual claims.

Moreover, in both experiments, competent children (i.e., in Experiment 1, children who evaluated the harm-related speech act more negatively than the no harm-related one, and in Experiment 2, children who evaluated the ill-intended speech act more negatively than the well-intended one, respectively) were more likely to use the appropriate justification type (consequences in Experiment 1, intentions in Experiment 2) rather than other justification categories than the other children (i.e., children who made the reverse evaluation or no difference between the puppets’ speech acts). These interrelations bolster the claim that children did not merely evaluate the incorrect factual claim *per se*, but focused on consequences and intentions, respectively. However, they also suggest that while as a group, children were competent at evaluating the factual claims in moral terms, there are also substantial individual differences in children’s competence for evaluation and justification that should be investigated in future work. We should also note that Experiment 2, in particular, was challenging regarding both the design [constant harm, incorrect speech act, (un)intended consequences] and the experimenter’s question which referred to the incorrectness of the factual claim (“X said it wrong. Is this mean, a little mean, good or very good of him?”). This might have led some children to focus on whether the assertion matched reality or not (thus not on moral questions). Similarly, Nobes and colleagues (2016) found that the phrasing of the experimenter’s question had a huge influence on children’s moral evaluation, such as whether they focused on intention or outcome. Moreover, the fact that the anticipated outcome would always be harmful in Experiment 2 (actual harm did not occur in the test phase) might in part explain why children’s evaluation in Experiment 2 was overall rather negative. Thus, future research could vary the intentionality of consequences while keeping anticipated consequences harmless.

The forced-choice test in Experiment 2 in which the clear majority of children correctly identified the puppet with ill intentions (and often referred to intentions in their reasoning) as the “mean” one supports the notion that preschoolers appreciate others’ intentions as morally relevant and use them for making moral evaluations. Similarly, Killen et al. (2011) found that from around late preschool age, children consider others’ intentions regarding morally relevant non-verbal actions in which an accidental transgressor caused harm. Given that Killen and colleagues found systematic associations between children’s competence in false belief tasks and their moral evaluation of the non-verbal actions, one interesting question for future research is whether theory of mind skills and moral evaluation of verbal actions – assertions underlain by good or bad intentions – are related.

Together, the present findings suggest that preschoolers’ normative understanding is not only confined to evaluating others’ intrinsically harmful (non-)verbal actions but also entails an appreciation of the moral dimension of factual claims that are typically merely true or false. And when children evaluate factual claims regarding their moral worth, they take into account consequences and intentions regarding consequences. The current work thus broadens the investigation of the ontogeny of normativity by integrating moral cognition with children’s developing understanding of speech acts, such as factual claims. Developing the ability to scrutinize and evaluate factual claims with moral relevance is a crucial skill, perhaps even more so in our digital age in which children are confronted with assertions in virtual forums on a daily basis.

## Ethics Statement

This research was conducted in accordance with the Declaration of Helsinki, the Ethical Principles of the German Psychological Society (DGPs), and the American Psychological Association (APA) but was not individually reviewed by the ethics committee as this is not obligatory at LMU Munich. It involved no invasive or otherwise ethically problematic techniques and no deception. All parents provided written informed consent, and children gave oral consent to participate in accordance with the Declaration of Helsinki.

## Author Contributions

MS supervised and provided funding for the project. MS developed the study concept. EF and MS designed the study. EF conducted the study and analyzed the data. Both authors interpreted the results. EF drafted the manuscript, and MS provided critical revisions. Both authors approved the final version of the manuscript for submission.

## Conflict of Interest Statement

The authors declare that the research was conducted in the absence of any commercial or financial relationships that could be construed as a potential conflict of interest.
